# Supplementary material for: Combining echocardiography with carotid ultrasound parameters in predicting major adverse cardiovascular events among older individuals with coronary artery disease
Source: PeerJ. 2025 Jul 23;13:e19688. doi: 10.7717/peerj.19688 (PMC12296572; doi:10.7717/peerj.19688)
Supplement: Supplemental Information 3 [file peerj-13-19688-s003.docx]

STROBE Statement—checklist of items that should be included in reports of observational studies

|  | Item No. | Recommendation | Page  No. | Relevant text from manuscript |
| --- | --- | --- | --- | --- |
| **Title and abstract** | 1 | (*a*) Indicate the study’s design with a commonly used term in the title or the abstract | 2 | A total of 138 elderly  individuals diagnosed with coronary heart disease and admitted to our facility from June  2019 to June 2020 were included in this study. These patients were categorized into two  groups: a non-MACE group consisting of 94 patients and a MACE group with 44 patients,  based on whether they experienced Major Adverse Cardiovascular Events (MACE) within  three years following their discharge. Additionally, a validation cohort of 141 patients was  assembled, which was also divided into a non-MACE group with 90 patients and a MACE  group with 51 patients. Upon admission, all participants underwent both transthoracic  echocardiography and carotid ultrasound assessments. We then conducted a comparative  analysis of the ultrasound parameters between the two groups. |
|  |  | (*b*) Provide in the abstract an informative and balanced summary of what was done and what was found | 2 | The MACE group  had signiûcantly higher left ventricular end-diastolic diameter (LVEDD), carotid intima-  media thickness (IMT), and Crouse score, and signiûcantly lower left ventricular ejection  fraction (LVEF) and peak velocity of diastolic late ûlling wave A compared to the non-MACE  group (P < 0.05). LVEDD, IMT, and Crouse score were risk factors for MACE, while LVEF and  A value were protective factors against MACE. The area under the curve (AUC) for the  combined model of MACE was 0.755. In the validation set, an AUC of 0.754 was obtained  for the combined model. |
| Introduction | | | |  |
| Background/rationale | 2 | Explain the scientific background and rationale for the investigation being reported | 4 | Coronary heart disease is a prevalent cardiovascular condition among older adults, typically presenting with symptoms like discomfort or tightness in the chest, pain in the chest area, and difficulties in breathing [1-3] .  38 Percutaneous coronary intervention (PCI) serves as the main approach for treating coronary heart disease, but  39 post-PCI, patients are prone to myocardial ischemia-reperfusion, leading to major adverse cardiovascular events  40 (MACE), which significantly affect the prognosis of coronary heart disease patients and increase their  41 readmission rates and mortality [4, 5] . Consequently, it is imperative to discover proactive indicators that can  42 forecast the severity and outcome of coronary heart disease. This proactive approach would facilitate early  43 interventions aimed at diminishing the likelihood of experiencing MACE. |
| Objectives | 3 | State specific objectives, including any prespecified hypotheses | 4 | Ultrasound is a convenient, non-invasive, and highly repeatable imaging tool [6-8] . Cardiac ultrasound can  45 observe abnormal movements of the ventricular walls and assess the extent of these abnormalities, thereby  46 reflecting the degree of myocardial ischemia and the extent of myocardial involvement in coronary artery  47 disease [9] . The carotid arteries are located superficially in the neck and serve as important conduits connecting  48 the heart and brain. Carotid ultrasound is an essential parameter for evaluating the degree of arterial stenosis and  49 the progression of atherosclerosis, and it has positive implications for guiding cardiovascular diseases [10-12] .  50 Currently, many studies both domestically and internationally use cardiac ultrasound and carotid ultrasound to  51 evaluate cardiac function and carotid atherosclerotic lesions, and their diagnostic value has been proven [13, 14] .  52 However, there are no reports on using cardiac ultrasound and carotid ultrasound to predict MACE in patients  53 with coronary heart disease. Therefore, this study aims to investigate the predictive accuracy of combining  54 cardiac ultrasound with carotid ultrasound examination parameters for predicting MACE in elderly patients with  55 coronary heart disease, providing new insights into the prediction methods for MACE in coronary heart disease  56 patients. |
| Methods | | | |  |
| Study design | 4 | Present key elements of study design early in the paper | 4 | This study retrospectively analyzed coronary heart disease patients hospitalized for treatment between June  61 2019 and June 2020. Individuals who fulfilled the specified inclusion and exclusion criteria were chosen as  62 participants for the study. Inclusion criteria: (1) Patients aged g60 years with chest pain symptoms within 24  63 hours; (2) Individuals who exhibited chest pain and were diagnosed with either acute myocardial infarction or  64 unstable angina; (3) Patients with normal comprehension abilities willing to participate in follow-up after  65 discharge. Exclusion criteria: (1) Individuals lacking complete clinical information; (2) Lost-to-follow-up  66 patients; (3) Patients transferred to another hospital for treatment after diagnosis; (4) Patients with chest pain  67 caused by other reasons such as trauma, generalized pain, rheumatic diseases; (5) Patients with comorbid  68 immune, hematological diseases, hepatitis, pulmonary infections, psychiatric disorders, malignant tumors.  69 This study was approved by Yantai Yuhuangding Hospital's ethics committee. As the study did not involve  70 human intervention, the ethics committee waived the patient's informed consent. |
| Setting | 5 | Describe the setting, locations, and relevant dates, including periods of recruitment, exposure, follow-up, and data collection | 4 | This study retrospectively analyzed coronary heart disease patients hospitalized for treatment between June  61 2019 and June 2020. Individuals who fulfilled the specified inclusion and exclusion criteria were chosen as  62 participants for the study. Inclusion criteria: (1) Patients aged g60 years with chest pain symptoms within 24  63 hours; (2) Individuals who exhibited chest pain and were diagnosed with either acute myocardial infarction or  64 unstable angina; (3) Patients with normal comprehension abilities willing to participate in follow-up after  65 discharge. Exclusion criteria: (1) Individuals lacking complete clinical information; (2) Lost-to-follow-up  66 patients; (3) Patients transferred to another hospital for treatment after diagnosis; (4) Patients with chest pain  67 caused by other reasons such as trauma, generalized pain, rheumatic diseases; (5) Patients with comorbid  68 immune, hematological diseases, hepatitis, pulmonary infections, psychiatric disorders, malignant tumors.  69 This study was approved by Yantai Yuhuangding Hospital's ethics committee. As the study did not involve  70 human intervention, the ethics committee waived the patient's informed consent. |
| Participants | 6 | (*a*) *Cohort study*—Give the eligibility criteria, and the sources and methods of selection of participants. Describe methods of follow-up  *Case-control study*—Give the eligibility criteria, and the sources and methods of case ascertainment and control selection. Give the rationale for the choice of cases and controls  *Cross-sectional study*—Give the eligibility criteria, and the sources and methods of selection of participants | 4 | This study retrospectively analyzed coronary heart disease patients hospitalized for treatment between June  61 2019 and June 2020. Individuals who fulfilled the specified inclusion and exclusion criteria were chosen as  62 participants for the study. Inclusion criteria: (1) Patients aged g60 years with chest pain symptoms within 24  63 hours; (2) Individuals who exhibited chest pain and were diagnosed with either acute myocardial infarction or  64 unstable angina; (3) Patients with normal comprehension abilities willing to participate in follow-up after  65 discharge. Exclusion criteria: (1) Individuals lacking complete clinical information; (2) Lost-to-follow-up  66 patients; (3) Patients transferred to another hospital for treatment after diagnosis; (4) Patients with chest pain  67 caused by other reasons such as trauma, generalized pain, rheumatic diseases; (5) Patients with comorbid  68 immune, hematological diseases, hepatitis, pulmonary infections, psychiatric disorders, malignant tumors.  69 This study was approved by Yantai Yuhuangding Hospital's ethics committee. As the study did not involve  70 human intervention, the ethics committee waived the patient's informed consent. |
|  |  | (*b*) *Cohort study*—For matched studies, give matching criteria and number of exposed and unexposed  *Case-control study*—For matched studies, give matching criteria and the number of controls per case |  |  |
| Variables | 7 | Clearly define all outcomes, exposures, predictors, potential confounders, and effect modifiers. Give diagnostic criteria, if applicable | 5 | On the second day of their hospital stay, patients underwent ultrasound assessments while positioned on  74 their left side and engaging in relaxed breathing. A color Doppler ultrasound device (m9cv, Mindray, China)  75 operating at a probe frequency of 2.0-4.0 MHz was employed to systematically capture images in various views,  76 including the parasternal short-axis, long-axis view of the left ventricle, two-chamber view, four-chamber apical  77 view, and short-axis view of the left ventricle. The data generated by the software analysis facilitated the  78 calculation of several metrics, including left ventricular end-systolic diameter (LVESD), left ventricular end-  79 diastolic diameter (LVEDD), left atrial dimension (LAD), left ventricular ejection fraction (LVEF), left  80 ventricular posterior wall thickness (LVPWT), interventricular septum thickness (IVST), the peak gradient of  81 the E-wave (E), the peak gradient of the A-wave (A), and the E/A ratio was subsequently derived. Positive  82 criteria for color Doppler ultrasound were defined as abnormal ultrasound cardiogram findings, including  83 abnormal motion of the diseased wall relative to the opposite wall, absence of left ventricular wall segmental  84 motion, localized hypokinesis, dyskinesis, and abnormal thickening during systole.  85 2.2.2 Carotid Artery Ultrasound  86 Patients were positioned supine with the neck fully exposed. The probe frequency was adjusted to 7.5-10.0  87 MHz. The probe was moved along the direction of the common carotid artery, between the sternocleidomastoid  88 muscle and the trachea, scanning continuously from top to bottom to display the proximal, distal, and mid-  89 sections of the common carotid artery. Subsequently, the probe was gently shifted from the anterior aspect of  90 the neck to the posterior region, traversing the carotid bifurcation in order to obtain images of the carotid arteries'  91 long axis, showcasing both the external and internal carotid arteries. Measurements were taken of the internal  92 carotid artery's lumen diameter, while the echo intensity of the arterial wall was evaluated. Additionally, an  93 assessment was conducted to identify any plaques, stenosis, or other morphological irregularities present within  94 the lumen. For severely narrowed or occluded vessels, collateral circulation in the distal vessel was noted. The  95 shape, size, distribution, and echo intensity of atherosclerotic plaques were observed. The direction and velocity  96 of blood flow, the presence of any flow defects or interruptions, and the location and degree of arterial stenosis  97 were observed. Intima-media thickness (IMT) was recorded. Parameters related to blood flow were documented,  98 encompassing diastolic velocity (Vd), systolic velocity (Vs), and the resistance index (RI). Positive criteria for  99 carotid ultrasound were defined as follows: IMT f1.0 mm as normal, 1.0 mm < IMT g 1.2 mm as carotid  100 atherosclerosis, and IMT > 1.2 mm as local plaque formation. The Crouse score was calculated: IMT f1.2 mm  101 as 0 points, 1.2 mm f IMT < 2.0 mm as 1 point, 2.1 mm f IMT < 4.0 mm as 2 points, and IMT g 4.1 mm as 3  102 points. |
| Data sources/ measurement | 8* | For each variable of interest, give sources of data and details of methods of assessment (measurement). Describe comparability of assessment methods if there is more than one group | *5-6* | *Following their discharge, patients were monitored every two months through phone calls and routine*  *105 outpatient visits. The follow-up period extended to three years to observe the incidence of major adverse*  *106 cardiovascular events (MACE) during this timeframe. In this research, MACE was defined to include non-fatal*  *107 myocardial infarction, angina pectoris, arrhythmia, heart failure, and all-cause mortality that occurred throughout*  *108 the follow-up interval. Based on whether MACE events took place, participants were categorized into a non- MACE group (n=84) and a MACE group (n=54). Additionally, a separate validation cohort of 141 patients was*  *110 established, which was also divided into a non-MACE group (n=90) and a MACE group (n=51). All patients*  *111 included in this study cooperated with the follow-up, and their relevant data were complete.* |
| Bias | 9 | Describe any efforts to address potential sources of bias | 6 | The statistical analysis was conducted utilizing SPSS version 26.0 (SPSS Inc., Chicago, IL, USA).  114 Continuous data that followed a normal distribution were reported as mean ± standard deviation and assessed  115 between groups using the independent samples t-test. Categorical variables were represented as n (%) and  116 analyzed for differences between groups via the chi-square (Ç²) test. A P-value of less than 0.05 was deemed  117 statistically significant. To evaluate the relationship between MACE and ultrasound parameters, Spearman  118 correlation analysis was employed. Variables demonstrating significant differences in both differential and  119 correlation assessments were factored in as covariates in the logistic regression analysis. Additionally, the area  120 under the receiver operating characteristic (ROC) curve (AUC) was utilized to evaluate the predictive capability  121 of ultrasound parameters concerning MACE. |
| Study size | 10 | Explain how the study size was arrived at | 5-6 | Following their discharge, patients were monitored every two months through phone calls and routine  105 outpatient visits. The follow-up period extended to three years to observe the incidence of major adverse  106 cardiovascular events (MACE) during this timeframe. In this research, MACE was defined to include non-fatal  107 myocardial infarction, angina pectoris, arrhythmia, heart failure, and all-cause mortality that occurred throughout  108 the follow-up interval. Based on whether MACE events took place, participants were categorized into a non- MACE group (n=84) and a MACE group (n=54). Additionally, a separate validation cohort of 141 patients was  110 established, which was also divided into a non-MACE group (n=90) and a MACE group (n=51). All patients  111 included in this study cooperated with the follow-up, and their relevant data were complete. |

Continued on next page

| Quantitative variables | 11 | Explain how quantitative variables were handled in the analyses. If applicable, describe which groupings were chosen and why | 6 | The statistical analysis was conducted utilizing SPSS version 26.0 (SPSS Inc., Chicago, IL, USA).  114 Continuous data that followed a normal distribution were reported as mean ± standard deviation and assessed  115 between groups using the independent samples t-test. Categorical variables were represented as n (%) and  116 analyzed for differences between groups via the chi-square (Ç²) test. A P-value of less than 0.05 was deemed  117 statistically significant. To evaluate the relationship between MACE and ultrasound parameters, Spearman  118 correlation analysis was employed. Variables demonstrating significant differences in both differential and  119 correlation assessments were factored in as covariates in the logistic regression analysis. Additionally, the area  120 under the receiver operating characteristic (ROC) curve (AUC) was utilized to evaluate the predictive capability  121 of ultrasound parameters concerning MACE. |
| --- | --- | --- | --- | --- |
| Statistical methods | 12 | (*a*) Describe all statistical methods, including those used to control for confounding | 6 | The statistical analysis was conducted utilizing SPSS version 26.0 (SPSS Inc., Chicago, IL, USA).  114 Continuous data that followed a normal distribution were reported as mean ± standard deviation and assessed  115 between groups using the independent samples t-test. Categorical variables were represented as n (%) and  116 analyzed for differences between groups via the chi-square (Ç²) test. A P-value of less than 0.05 was deemed  117 statistically significant. To evaluate the relationship between MACE and ultrasound parameters, Spearman  118 correlation analysis was employed. Variables demonstrating significant differences in both differential and  119 correlation assessments were factored in as covariates in the logistic regression analysis. Additionally, the area  120 under the receiver operating characteristic (ROC) curve (AUC) was utilized to evaluate the predictive capability  121 of ultrasound parameters concerning MACE. |
|  |  | (*b*) Describe any methods used to examine subgroups and interactions | 6 | The statistical analysis was conducted utilizing SPSS version 26.0 (SPSS Inc., Chicago, IL, USA).  114 Continuous data that followed a normal distribution were reported as mean ± standard deviation and assessed  115 between groups using the independent samples t-test. Categorical variables were represented as n (%) and  116 analyzed for differences between groups via the chi-square (Ç²) test. A P-value of less than 0.05 was deemed  117 statistically significant. To evaluate the relationship between MACE and ultrasound parameters, Spearman  118 correlation analysis was employed. Variables demonstrating significant differences in both differential and  119 correlation assessments were factored in as covariates in the logistic regression analysis. Additionally, the area  120 under the receiver operating characteristic (ROC) curve (AUC) was utilized to evaluate the predictive capability  121 of ultrasound parameters concerning MACE. |
|  |  | (*c*) Explain how missing data were addressed | 6 | The statistical analysis was conducted utilizing SPSS version 26.0 (SPSS Inc., Chicago, IL, USA).  114 Continuous data that followed a normal distribution were reported as mean ± standard deviation and assessed  115 between groups using the independent samples t-test. Categorical variables were represented as n (%) and  116 analyzed for differences between groups via the chi-square (Ç²) test. A P-value of less than 0.05 was deemed  117 statistically significant. To evaluate the relationship between MACE and ultrasound parameters, Spearman  118 correlation analysis was employed. Variables demonstrating significant differences in both differential and  119 correlation assessments were factored in as covariates in the logistic regression analysis. Additionally, the area  120 under the receiver operating characteristic (ROC) curve (AUC) was utilized to evaluate the predictive capability  121 of ultrasound parameters concerning MACE. |
|  |  | (*d*) *Cohort study*—If applicable, explain how loss to follow-up was addressed  *Case-control study*—If applicable, explain how matching of cases and controls was addressed  *Cross-sectional study*—If applicable, describe analytical methods taking account of sampling strategy | 6 | The statistical analysis was conducted utilizing SPSS version 26.0 (SPSS Inc., Chicago, IL, USA).  114 Continuous data that followed a normal distribution were reported as mean ± standard deviation and assessed  115 between groups using the independent samples t-test. Categorical variables were represented as n (%) and  116 analyzed for differences between groups via the chi-square (Ç²) test. A P-value of less than 0.05 was deemed  117 statistically significant. To evaluate the relationship between MACE and ultrasound parameters, Spearman  118 correlation analysis was employed. Variables demonstrating significant differences in both differential and  119 correlation assessments were factored in as covariates in the logistic regression analysis. Additionally, the area  120 under the receiver operating characteristic (ROC) curve (AUC) was utilized to evaluate the predictive capability  121 of ultrasound parameters concerning MACE. |
|  |  | (*e*) Describe any sensitivity analyses | NA | NA |
| Results | | | | |
| Participants | 13* | (a) Report numbers of individuals at each stage of study—eg numbers potentially eligible, examined for eligibility, confirmed eligible, included in the study, completing follow-up, and analysed | 6 | This study ultimately included 138 coronary heart disease patients who met the inclusion criteria. The 3-  126 year follow-up results after discharge showed that out of the 138 patients, 54 (39.13%) experienced MACE,  127 including 18 all-cause deaths (13.04%), 14 non-fatal myocardial infarctions (10.14%), 9 cases of angina pectoris  128 (6.52%), 7 cases of arrhythmia (5.07%), and 6 cases of heart failure (4.35%) (Table 1). Based on different  129 prognoses during the follow-up period, patients were divided into a non-MACE group (n=84, 60.87%) and a  130 MACE group (n=54, 39.13%). |
|  |  | (b) Give reasons for non-participation at each stage | NA | NA |
|  |  | (c) Consider use of a flow diagram | NA | NA |
| Descriptive data | 14* | (a) Give characteristics of study participants (eg demographic, clinical, social) and information on exposures and potential confounders | 6 | Table 2 presents the baseline characteristics of both patient groups. The findings indicate that there are no  133 statistically significant differences in the baseline information between these groups (P > 0.05) (Table 2),  134 suggesting that demographic and disease-related characteristics at baseline do not substantially influence the  135 occurrence of MACE. |
|  |  | (b) Indicate number of participants with missing data for each variable of interest | NA | NA |
|  |  | (c) *Cohort study*—Summarise follow-up time (eg, average and total amount) | 6 | This study ultimately included 138 coronary heart disease patients who met the inclusion criteria. The 3-  126 year follow-up results after discharge showed that out of the 138 patients, 54 (39.13%) experienced MACE,  127 including 18 all-cause deaths (13.04%), 14 non-fatal myocardial infarctions (10.14%), 9 cases of angina pectoris  128 (6.52%), 7 cases of arrhythmia (5.07%), and 6 cases of heart failure (4.35%) (Table 1). Based on different  129 prognoses during the follow-up period, patients were divided into a non-MACE group (n=84, 60.87%) and a  130 MACE group (n=54, 39.13%). |
| Outcome data | 15* | *Cohort study*—Report numbers of outcome events or summary measures over time | *6* | *This study ultimately included 138 coronary heart disease patients who met the inclusion criteria. The 3-*  *126 year follow-up results after discharge showed that out of the 138 patients, 54 (39.13%) experienced MACE,*  *127 including 18 all-cause deaths (13.04%), 14 non-fatal myocardial infarctions (10.14%), 9 cases of angina pectoris*  *128 (6.52%), 7 cases of arrhythmia (5.07%), and 6 cases of heart failure (4.35%) (Table 1). Based on different*  *129 prognoses during the follow-up period, patients were divided into a non-MACE group (n=84, 60.87%) and a*  *130 MACE group (n=54, 39.13%).* |
|  |  | *Case-control study—*Report numbers in each exposure category, or summary measures of exposure | *NA* | *NA* |
|  |  | *Cross-sectional study—*Report numbers of outcome events or summary measures | *NA* | *NA* |
| Main results | 16 | (*a*) Give unadjusted estimates and, if applicable, confounder-adjusted estimates and their precision (eg, 95% confidence interval). Make clear which confounders were adjusted for and why they were included | 7 | Using the occurrence of MACE as the dependent variable (coded as occurrence = 1, non-occurrence = 0),  148 a multivariate logistic regression analysis was conducted with the cardiac ultrasound parameters (LVEDD,  149 LVEF, and A value) and carotid ultrasound parameters (IMT and Crouse score, all considered as actual  150 measurement values) as independent variables. The analysis revealed that LVEDD, IMT, and Crouse score were  151 identified as risk factors for MACE in elderly individuals with coronary heart disease (OR > 1), whereas LVEF  152 and A value were found to be protective factors against the occurrence of MACE (OR < 1) (Table 5). |
|  |  | (*b*) Report category boundaries when continuous variables were categorized | NA | NA |
|  |  | (*c*) If relevant, consider translating estimates of relative risk into absolute risk for a meaningful time period | 7 | Using the occurrence of MACE as the dependent variable (coded as occurrence = 1, non-occurrence = 0),  148 a multivariate logistic regression analysis was conducted with the cardiac ultrasound parameters (LVEDD,  149 LVEF, and A value) and carotid ultrasound parameters (IMT and Crouse score, all considered as actual  150 measurement values) as independent variables. The analysis revealed that LVEDD, IMT, and Crouse score were  151 identified as risk factors for MACE in elderly individuals with coronary heart disease (OR > 1), whereas LVEF  152 and A value were found to be protective factors against the occurrence of MACE (OR < 1) (Table 5). |

Continued on next page

| Other analyses | 17 | Report other analyses done—eg analyses of subgroups and interactions, and sensitivity analyses | 7 | 3.6 Predictive value of combining cardiac ultrasound with carotid ultrasound for MACE in elderly  154 patients with coronary heart disease  155 The area under the ROC curve for the combined prediction of MACE using LVEDD, LVEF, A-value, IMT,  156 and Crouse integral was 0.755 (Fig.1), indicating good predictive value for MACE in elderly patients with  157 coronary heart disease when combining cardiac ultrasound with carotid ultrasound.  158 3.7 General Information (Validation Set)  159 In the validation set, 90 patients did not experience MACE, while 51 patients did. There were no significant  160 differences in baseline information between the two groups (P > 0.05) (Table 6), suggesting that other factors  161 may have influenced the occurrence of MACE.  162 3.8 Predictive Indicators (Validation Set)  163 In the validation set, cardiac ultrasound parameters and carotid ultrasound parameters also showed  164 significant differences. Among them, the MACE group had significantly higher LVEDD, IMT, and Crouse  165 integral and significantly lower LVEF and A-value compared to the non-MACE group (P < 0.05) (Table 7). This  166 indicates that these indicators have a significant impact on the occurrence of MACE.  167 3.9 Joint Model (Validation Set)  168 A combined predictive model for MACE was constructed using cardiac ultrasound parameters and carotid  169 ultrasound parameters. The results showed that the AUC of the combined model in the validation set was  170 0.754(Fig. 2), indicating good predictive value for the occurrence of MACE using the combination of cardiac  171 ultrasound and carotid ultrasound parameters. |
| --- | --- | --- | --- | --- |
| Discussion | | | | |
| Key results | 18 | Summarise key results with reference to study objectives | 8 | Coronary heart disease (CHD) progresses over time, affecting patients throughout their lifetime and is a  175 leading cause of death globally [15-17] . The prognosis of CHD patients is closely related to the number of diseased  176 coronary arteries and the severity of lesions. Early diagnosis and intervention are critical for improving  177 prognosis, but currently, there is a lack of specific markers for assessing the prognosis of CHD. This study aimed  178 to evaluate the predictive accuracy of a combined model using cardiac and carotid ultrasound parameters for  179 MACE in elderly CHD patients. Our results indicate that the combination of LVEDD, LVEF, A value, IMT, and  180 Crouse integral has good predictive value for MACE in this patient population. |
| Limitations | 19 | Discuss limitations of the study, taking into account sources of potential bias or imprecision. Discuss both direction and magnitude of any potential bias | 9 | While this study offers valuable new perspectives, it is important to recognize certain limitations. Primarily,  218 being a single-center retrospective study with a limited sample size may lead to selection bias; therefore, one  219 must interpret the findings with caution. Additionally, the follow-up period was limited to 3 years, preventing  220 the evaluation of long-term outcomes. To obtain more meaningful results, further studies should increase the  221 sample size and design prospective clinical trials for validation |
| Interpretation | 20 | Give a cautious overall interpretation of results considering objectives, limitations, multiplicity of analyses, results from similar studies, and other relevant evidence | 9 | In summary, this study suggests that a combination of cardiac and carotid ultrasound parameters can predict  225 MACE within 3 years in elderly CHD patients. Through cardiac and carotid ultrasound examination, not only  226 can the diagnostic rate of cardiovascular diseases be improved, but the prognosis of CHD patients can also be  227 predicted, providing objective evidence for clinicians to identify high-risk CHD patients early. |
| Generalisability | 21 | Discuss the generalisability (external validity) of the study results | 9 | In summary, this study suggests that a combination of cardiac and carotid ultrasound parameters can predict  225 MACE within 3 years in elderly CHD patients. Through cardiac and carotid ultrasound examination, not only  226 can the diagnostic rate of cardiovascular diseases be improved, but the prognosis of CHD patients can also be  227 predicted, providing objective evidence for clinicians to identify high-risk CHD patients early. |
| Other information | |  | | |
| Funding | 22 | Give the source of funding and the role of the funders for the present study and, if applicable, for the original study on which the present article is based | NA | NA |

*Give information separately for cases and controls in case-control studies and, if applicable, for exposed and unexposed groups in cohort and cross-sectional studies.

**Note:** An Explanation and Elaboration article discusses each checklist item and gives methodological background and published examples of transparent reporting. The STROBE checklist is best used in conjunction with this article (freely available on the Web sites of PLoS Medicine at http://www.plosmedicine.org/, Annals of Internal Medicine at http://www.annals.org/, and Epidemiology at http://www.epidem.com/). Information on the STROBE Initiative is available at www.strobe-statement.org.
